# Analytical Investigation of *Cymbopogon citratus* and Exploiting the Potential of Developed Silver Nanoparticle Against the Dominating Species of Pathogenic Bacteria

**DOI:** 10.3389/fmicb.2019.00282

**Published:** 2019-02-27

**Authors:** Priyanka Basera, Meeta Lavania, Anil Agnihotri, Banwari Lal

**Affiliations:** The Energy and Resources Institute, New Delhi, India

**Keywords:** *Cymbopogon citratus*, analytical analysis, *Cymbopogon citratus* silver nanoparticles, pathogenic species, minimum inhibitory concentration

## Abstract

Indian biodiversity is a hub for medicinal plants. Extensive research has been carried out to select plants with numerous properties which can be used for human welfare. Present research is about *Cymbopogon citratus*, an economically valuable medicinal plant. In this study *Cymbopogon citratus* was elected as a subject plant over the five selected plants (*Azadirachta indica*, *Plumeria obtuse*, *Sapindus mukorossi*, *Capsicum annuum* and *Phyllanthus emblica*) on the basis of antibacterial effect against dominating pathogenic species of gram positive (*Bacillus cereus, Bacillus licheniformis*) and gram negative (*Pseudomonas aeruginosa, Escherichia coli*) bacteria. Further, bioactive agents behind antibacterial potential of *Cymbopogon citratus* was analyzed using analytical method (Phyto-chemical, FTIR, NMR and GC-MS). Due to the broad antimicrobial spectrum, silver nanoparticles have turned into a noteworthy decision for the improvement of new medication. Therefore, this investigation further elaborated in the development of *Cymbopogon citratus* silver nano-particles (CNPs). Antibacterial potential of CNPs examine in a range of C_25_–C_150_ (μg/ml) through minimum inhibitory concentration (MIC) and minimum bactericidal concentration (MBC) where, C_25_ (μg/ml) concentration of CNPs were recorded as the MIC for all bacterial species and C_25_ (μg/ml) and C_50_ (μg/ml) noted as the MBC for *Pseudomonas aeruginosa, Escherichia coli* and *Bacillus cereus, Bacillus licheniformis*, respectively. In agar disk diffusion assay of CNPs, maximum diameter of zone of inhibition was observed for C_150_ (μg/ml) concentration *Bacillus cereus* (20.12 ± 0.42), *Bacillus licheniformis* (22.34 ± 0.4), *Pseudomonas aeruginosa* (35.23 ± 0.46) and *Escherichia coli* (31.87 ± 0.24). Involvement of bioactive component as a reducing and capping agent can be confirmed through FTIR spectrum of CNPs. Moreover XRD, EDXRF and SEM showed crystalline and cuboidal nature of CNPs with _∼_35 nm sizes. Prominently, cytotoxic analysis was conducted to understand the toxic effect of CNPs. This research highlights the potential of CNPs due to the bioactive components present in *Cymbopogon citratus* extract: Polyphenols (phenol; 1584.56 ± 16.32 mg/L, Flavanoids) and mixture of terpenoids (Citral, Myrcene, Farnesol, β-myrcene and β –Pinene)

## Introduction

More than half of the world population use plants for their basic health needs. They are bestowed for humanity. From the millennia they have been used as a potent medicinal treatment against various kinds of diseases and ailment. Plant resides wide range of bioactive components/secondary metabolites viz Phenols, Tannins, Saponin, Steriods, Alkaloids, Flavanoids, Carbohydrates and Glycosides which are responsible for antimicrobial activity (Nascimento et al., 2000; Ewansiha et al., 2012; Chaudhary et al., 2017; Elish et al., 2017) therefore the discovery of extraction of bioactive components from plants have proved to be one of the important research for human kind. Due to the negative impact of chemically designed antimicrobial drugs as compared to natural drugs on human health and environment; treatment with natural constituents has increased potentially these days (Wu et al., 2013). Many reports cited numerous plants with antimicrobial activity; *C. citratus* is one of them commonly referred as lemon grass.

*C*ymbopogon *citratus* is an aromatic, perennial and economically valuable plant. Studies indicate that this plant have a strong lemon like aroma due to the presence of citral, which is a potent bioactive constituent having antimicrobial activity (Korenblum et al., 2013) and therefore in common language, *C. citratus* called by lemongrass. The component present in plant is conventionally used in variety of human therapy (Han and Parker, 2017). Traditionally aqueous extract of its dried leaves use to treat digestive disorders, diabetes, nervous disorders and cancer (Francisco et al., 2011). According Thangam et al., 2014 findings *C. citratus* polysaccharides has important role in turning off the genes that suppresses the tumor growth and act as a potent novel anti-cancer drugs. *C. citratus*is widely applicable in field of medicine, cosmetic (Perfumes, soap etc) and brewing (non-alcoholic like tea) (Ekpenyong et al., 2014). Because of the immense benefit to human kind, *C. citratus* has been used to conduct several of studies for investigation of its valuable potential.

Different physio-chemical methods have been opted for synthesizing nanoparticles with expensive cost and detrimental effects (Oves et al., 2013). Recently, production of plant nanoparticles is in high demand; due to the versatile nature of plant to be used as an antimicrobial agent. Attention of researchers has been diverted toward plants nanoparticle (PNPs) because of their eco-friendly and non-pathogenic nature. They have provided an environmentally suitable solution in bio-medicinal sector, as a green approach (Jain and Mehata, 2017; Oves et al., 2018). In general; a particle which range between 1 and 100 nm is referred to as nanoparticle (Baranwal et al., 2018). Due to the reduced size of synthesized nanoparticles, high surface to volume ratio was noticed by scientist which is a remarkable feature for obliterating bacterial density (Oves et al., 2017). For the production of nanoparticles noble metals like Silver (Ag), Gold (Au) and Platinum (Pt) have been used (Rai et al., 2015). Silver (Ag) used enormously among all metals, as researcher reported its use in broad sense as an anti-microbial agent like anti-bacterial, anti-fungal, anti-inflammatory, anti-cancerous and anti-viral (Goudarzi et al., 2016; Oves et al., 2017; Qayyum et al., 2017; Yugal et al., 2017). Therefore synthesizing silver nanoparticle through plant extract has been recognized as efficient biological approach in controlling the pathogenic microbes (Qayyum et al., 2017).

The study has focused on (i) Screening of potent plant from six different selected plants (*Azadirachta indica, Plumeria obtuse, Cymbopogon citratus*, *Sapindus mukorossi*, *Capsicum annuum* and *Phyllanthus emblica*) using antibacterial assay against dominating pathogenic gram positive (*Bacillus cereus, Bacillus licheniformis*) and gram negative (*Pseudomonas aeruginosa, Escherichia coli*) bacterial species.(ii) Characterization of bioactive components present in selected potent plant (*Cymbopogon citratus*) through phytochemical (qualitatively and quantitatively) and analytical (FTIR, NMR and GC-MS) analysis. (iii) Development of synthesized nanoparticles from *Cymbopogon citratus* referred as CNPs and further analyzed using FTIR, XRD, EDXRF, SEM, MIC/MBC and cyto-toxicity assay.

## Materials and Methods

### Collection of Plant Material

Leaves of *A. indica, C. citrates*, and *P. obtuse* were collected from the institutional area of TERI-gram (Gurugram, India) in the month of June-July (2016). Seeds of *S. mukorossi, C. annuum*, and *P. emblica* obtained from raw fruits, commercially available. Study materials were stored at cold room (4^°^C) till further analysis.

### Preparation of Plant Extracts

To prepare plant extract, leaves and raw fruits were first surface sterilized with distilled water followed by 70% ethanol (Merck Ltd) for removal of dust and unwanted particles. Seeds were obtained from raw fruits by removing the pulp and sterilized with 70% ethanol (Merck, Ltd). Sterilized plant material (leaves and seeds) were sun-dried and then powdered with mechanical grinder. For extraction, soxhlet method was opted, were methanol (Fisher Scientific, Ltd) served as a solvent. For each plant, different conditions such as ratio of solvent (ml): plant material (g) and time were optimized. Extracted samples were further concentrated using Rota-evaporator (Rotavac Heidolph).

### Testing Microorganism

In the present study, for examining the antimicrobial activity; *B. cereus* (ATCC- BAA-512), *E. coli* (ATCC- 11775), *P. aeruginosa* (ATCC- 19429) and *B. licheniformis* (DSMZ-8059) bacteria were selected. For inoculum preparation loop full of bacterial culture from agar plates were transferred to Muller Hinton broth (HiMedia). Obtained bacterial suspension was incubated for 24 h at 37°C, further for antibacterial assay bacterial growth were adjusted to 0.5 MacFarland standard turbidity (Clinical and Laboratory Standards Institute [CLSI], 2012) approx 1.5 × 10^6^ CFU/ml bacterial cultures.

### Antibacterial Activity of Plant Extracts (Agar Disk-Diffusion)

For determination of antibacterial activity, agar disk-diffusion method was performed as per EUCAST (2019) Guidelines. Using Spread plate technique, Mueller-Hinton agar (HiMedia Laboratories Pvt. Ltd) plates were inoculated with selected microorganism. Sterile disk (6 mm diameter, HiMedia Laboratories Pvt., Ltd.) was dipped in different plant extracts (*Azadirachta indica, Plumeria obtuse, Cymbopogon citratus*, *Sapindu smukorossi*, *Capsicum annuum* and *Phyllanthus emblica*) solutions in selected concentration (100 μg/ml) made in methanol. Plates were incubated at 37°C for 24 h. The clear zone was noted in mm, which signifies the Zone of inhibition. From this investigation, *C. citratus* was selected for further examinations.

### Qualitative and Quantitative Analysis of *C. citratus* Extract

Bioactive compounds present in *C. citratus* extract were assessed both qualitatively and quantitatively (TPC: Total phenolic compound). The standard protocols were used for determination of compounds such as carbohydrate, Saponin, Steroid, Phenols, Tannin, Flavonoids, Glycosides, Terpenoids, and Alkaloids (Ewansiha et al., 2012). Total phenolic content of the plant extract was determined through Folin-Ciocalteu reagent (Alhakmani et al., 2013).500 μl extract (1 mg/mL) mixed with 2 ml of 10% Follin-Coiocalteu (2N) (Sigma-Aldrich) and 4 ml of 7.5%NaHCO_3_ (Sigma-Aldrich) solution. Mixture was incubated for 30 min at room temperature. Absorbance was measured at wavelength 765 nm. Standard curve (Supplementary Figure S1) was prepared using gallic acid. Total Phenolic content was expressed in mg/g of dry weight.

### Analytical Investigation of *C. citratus* Extract

#### Fourier Transform Infrared Spectroscopy (FTIR)

Fourier transform infrared spectroscopy spectrum of *C. citratus* extract was obtained using FTIR spectrophotometer (Perkin Elmer). FTIR used for chemical identification as each molecule and chemical structure creates a unique spectra. The IR spectra were accounted in % transmittance. The wave number region for analysis was 4000–400 cm^-1^ (mid-infrared range.) with resolution of 0.15 cm^-1^ having 64 scans per spectrum.

#### Nuclear Magnetic Resonance (NMR)

Nuclear magnetic resonance spectroscopy was performed to obtain ^1^H-NMR spectra of *C. citratus* extract, which was recorded using NMR (BrukerAvance III) spectrophotometer with operating frequency of 400 MHz at 289K temperature, the spectra obtained of 12 ppm width. To acquire high-quality spectra; sample run for 64 scans and chemical shifts were reported in parts per millions (ppm).

#### Gas Chromatography-Mass Spectrometry (GC-MS)

The extract of *C. citratus* was analyze using GC-MS (Agilent 5975C) equipped with DB-WAX capillary column. Helium was used as carrier gas. Temperature ranges between 230 and 325°C. Initially, column temperature was set at 70°C and further increased to 325°C. Dilute sample (1/50 in methanol) of 0.1 μl was used. The components were identified on the basis of their mass spectra using National Institute for Standards and Technology [NIST] Mass Spectrometry Data Center (2018) library data of GC-MS system.

### Development, Characterization and Surface Analysis of *C. citratus* Silver Nano-Particle (CNPs)

#### Preparation of Silver Nano-Particle

The nanoparticle synthesized from *C. citratus* was mentioned as CNPs. Silver nanoparticle was prepared by mixing plant extract in ratio of 1:9 10 ml plant extract and 90 ml of 1 mM (AR grade AgNO_3_, Sigma-Aldrich, India). This was incubated at 37°C for 24 h in dark condition. Colloidal suspension was obtained which was further centrifuged two times at 4000 rpm for 30 min; collected pellet was washed with distilled water with same condition for removal of any absorbed substances. The obtained nanoparticles were lyophilized and used as stock (Simon et al., 2017). From stock, working solution (in methanol) with varying range (C_25_-C_150_μg/ml) was prepared.

#### X-Ray Diffraction (XRD), Energy Dispersive X-Ray Fluorescence (EDXRF) and Fourier Transform Infrared Spectroscopy (FTIR) Analysis of CNPs

The prepared CNPs were characterized by; X-ray diffraction (XRD), energy dispersive X-ray fluorescence spectrometry (EDXRF) and Fourier transform infrared (FT-IR) spectroscopy. Analysis of lyophized powder of synthesized CNPs was carried out in a Rigaku Mini-Flex II XRD machine to determine the phase crystallinity using Cu-κα radiation. XRD analysis was executed with in the 2θ scanning range of 3°–90° with speed 4.00 deg/min in continuous mode. The EDXRF was executed using DX-700HS spectrometer (Shimadzu) for the purity of silver. The XRF study has been carried out in Helium atmosphere, at suitable voltage and current intensity. FT-IR spectra were recorded over the range of 400–4000 cm^-1^ using a FT-IR Perkin Elmer spectrophotometer.

#### Scanning Electron Microscopy (SEM) of CNPs

Interactions between bacterial species and silver nanoparticle were studied by Scanning Electron Microscopy (Carl Zeiss) (Hayat, 2000). Under aseptic conditions sample was immersed 2 to 4 h in 2.5% glutaraldehyde solution. Primary washing was done by 0.1M phosphate buffer with pH 7.2 and dehydrated with ethanol solution in series of 10–100% followed by acetone. Samples were air dried overnight, which were further coated with thin layer of metal (gold and palladium).

#### Minimum Inhibitory Concentration (MIC) and Minimum Bactericidal Concentration (MBC) of CNPs

Minimum inhibitory concentration were performed using broth dilution technique, value which shows the 99.9% of bacterial inhibition after 24 h of incubation at 37°C were considered as a MIC. For determination of MBC a small portion of liquid was aliquots from the MIC wells and spread on agar plates for 24 h at 37°C, no visible bacterial growth after sub-culturing was considered as MBC (French, 2006). Under aseptic condition MIC was conducted using Muller Hinton broth (MHB) medium, purchased from HiMedia Laboratories Pvt Ltd. Potential of CNPs were tested in a range from 25 to 150 μg/ml placing with positive and negative controls. Positive control was a bacterial suspension in MHB broth whereas negative controls were wells containing only MHB and MHB with CNPs. Experiment was incubated for 24 h at 37°C, appearance of visible solution in treated wells with reference to positive and negative control reflected as the MIC value of CNPs against test organism.

#### Cytotoxicity Assay of *C. citratus* and CNPs

The cytotoxicity assay was performed according to Ishida et al. (2006). The RBCs suspension was treated with several concentrations of both plant extract and synthesized CNPs in range of 10–150 μg/ml for 48 h where Triton X-114 (Sigma-Aldrich), utilized as a marker for hemolysis. Incubation executed for 2 h at 37°C on experimental cells. The RBCs were centrifuged at 2000 for 10 min, and estimation of hemolysis was done by taking the supernatant part, using a spectrophotometer at 540 nm. The outcomes were communicated as the percentage of hemolysis cytotoxicity test was conducted at National Toxicology Institute, Pune, India.

### Statistical Analysis

The experimental results were expressed as mean standard deviation of three replicates. Microsoft Excel 2010 statistical package was used for all analyses. The data were subjected to one-way analysis of variance (ANOVA) to determine the significant difference among variables. Difference was considered statistically significant at p ≤ 0.05.

## Results and Discussion

### Selection of Potent Plant on the Basis of Antibacterial Potential

**FIGURE 1:**
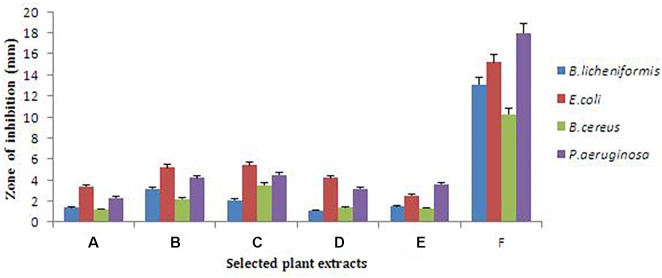
Antibacterial activity depicted in various plant extracts A (*Phyllanthus emblica*), B (*Azadirachta indica*), C (*Capsicum annuum*), D (*Sapindus mukorossi*), E (*Plumeria obtuse*), F (*Cymbopogoncitratus*). *Cymbopogon citratus* displayed highest antibacterial activity. Mean values are significantly differ from each other according to statistical one way ANOVA (*P* ≤ 0.05).

**FIGURE 2:**
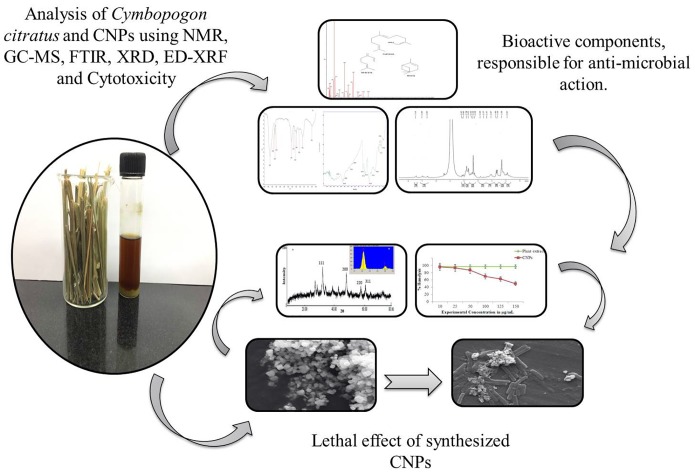
Schematic representation of analytical analysis of *Cymbopogon citratus* and lethal effect of synthesized silver nano-particles (CNPs).

Antimicrobial assay was performed to select the potent plant among the six selected plants. According to Ishida et al. (2006) plants inherent with medicinal properties which were studied for antimicrobial activities. Our finding signifies the importance of *C. citratus* among all selected plants; *C. citratus* gave the highest zone of inhibition against all the test microorganisms. The diameter of zone of inhibition recorded were 10.23 ± 0.08 for *B. cereus*, 13.1 ± 0.3 for *B. licheniformis*, 15.2 ± 0.5 for *E. coli* and 18.0 ± 0.12 for *P. aeruginosa* (Figure 1 and Supplementary Table S1). Therefore, *C. citratus* selected and subjected for further analysis in order to identify its bioactive components responsible for its antibacterial nature and potential of synthesized silver nanoparticle against test microorganisms (Figure 2).

### Qualitative and Quantitative Analysis

Previous reports of Matouschek and Stahl (1991), Chisowa et al. (1998), Negrelle and Gomes (2007), Aftab et al. (2011), and Lawal et al. (2017) on *C. citratus* suggested that geographical origin is responsible for variation in chemical composition of plant extract, though presence of sugar, steroid, phenols, tannins, alkaloid, flavanoid and terpenoid is well-known. Results of preliminary phytochemical screening confirmed the presence of similar chemical classes (Table 1). Existence of these chemicals in plant can act as bioactive components for plant, which are responsible for antimicrobial activity (Shah et al., 2011). Phenolic compounds are ubiquitous in nature and they exert several functions on plants such as such as plant growth, reproduction, development and disease resistance. These compounds were considered as the vital bioactive moiety for plant and produced by shikimate pathway (Lin et al., 2016). Several reports were suggesting that phenolic compound including terpenoid and flavonoid are anti-microbially bioactive components reside in plants (Parveen et al., 2010; Bhat et al., 2011; Voon et al., 2012; Wu et al., 2013). Quantitative finding of present research for the concentration of phenol was estimated to be 1584.56 ± 16.32 mg/g (Table 2). According to Mirghani et al. (2012) study, high amount of phenol is reported in *C. citratus*. Previously reported data on phenolic compounds including flavonoids validate that, these compounds contain polar hydroxyl groups which were responsible for antioxidants, free radical scavenger, anti-bacterial and anti-inflammatory actions (Cheel et al., 2005; Compean and Ynalvez, 2014; Pratiwi and Elin, 2016). Researcher additionally believed that inhibitory impact of bioactive component (terpenoids and phenols) is due to the interaction and disruption of enzymes and proteins useful for microbial metabolism (Ashraf et al., 2016).

**Table 1:** Qualitative analysis of phytochemical constituent present in *C. citratus* extract.

S. No.	Phytochemical test	Methanolic extract of *C. citratus*
1	Carbohydrates	+
2	Saponin	-
3	Steriod	+
4	Phenols	+++
5	Tannins	+
6	Flavanoid	++
7	Alkaloid	+
8	Terponoid	+++
9	Cardiac glycoside	+


*“+++” Highly present; “+” moderate present; and “–” absent.*

**Table 2:** Quantitative analysis of phytochemical constituent present in *C. citratus* extract.

Phytochemical test	mg/ggallic acid equivalent
Total phenolic content (TPC)	1584.56 ± 16.32


*±signify the Standard Deviation (SD) value.*

### Analytical Analysis

To obtain the reliable statistical data, analytical analysis of *C. citratus* was executed through FTIR, NMR and GC-MS; CNPs through XRD, EDXRF and FTIR. Further, comparative analysis of FTIR spectra of *C. citratus* and CNPs were illustrated.

### FTIR Analysis of *C. citratus* and CNPs

**FIGURE 3:**
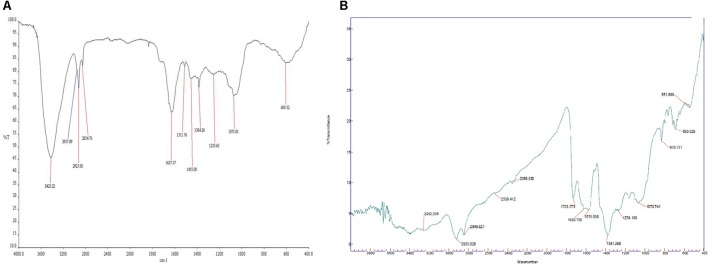
**(A)** FTIR spectra of *Cymbopogon citratus* (methanolic extract), and **(B)** FTIR of CNPs.

For analyzing surface functional groups in plant extract and CNPs, FTIR technique was used as represented in Figure 3A,B. The spectra obtained for *C. citratus* (plant) extract and CNPs exhibited well defined spectral regions varied in the range of 400–4000 cm^-1^. While comparing FTIR spectra of plant extract with CNPs, peaks of CNPs spectra observed at 693.529, 1070.741, 1381.988, 1620.7, 2849.921 and 2923.029 was found to be almost similar with minor shifts, indicates the role of plant extract as a capping agent to the silver nanoparticles (Ahmed and Ikram, 2015). According to Jain and Mehata (2017) plant extract contains the responsible agent for the reduction of silver ions into the formation of nanoparticles. Further, spectrum lying in range between 3600 and 3200 was assigned to -OH stretching of alcohol. The peaks in the region of 1700–1600 denotes the C = O and weak C = C stretching of aldehyde and ketone. C-H stretching of alkane was observed at 2923.029 (CNPs), 2925.80 (Plant extract) corresponding to an alkyl saturated aliphatic group. Peak at 2849.921 (CNPs), 2854.73 (Plant extract) related to symmetric and asymmetric stretching of CH_2_, presence of similar carbon stretch was reported by Wany et al. (2014) and Jamuna et al. (2017). Observed spectra within 1400–1000 range correspond to N-H stretch of amines (1° and 2°) and C-O stretch (groups of polyphenols like flavanoid, terpenoids and polysaccharides) (Vankar and Shukla, 2012). The FTIR results confirmed the presence of –N-H, –OH, C = C, and C-H groups, which indicated that the plant extract containing the hydroxyl and amine groups that evidence the presence of flavonoids and terpenoids. Further, presence of flavonoids was responsible for reducing Ag^+^ to Ag^0^ and an amino group stabilized the synthesized nanoparticles (Balashanmugam and Kalaichelvan, 2015). Overall, biological agent resides in plants are responsible for capping and stabilizing the synthesized silver nanoparticles.

### ^1^H-NMR Analysis of *C. citratus*

**FIGURE 4:**
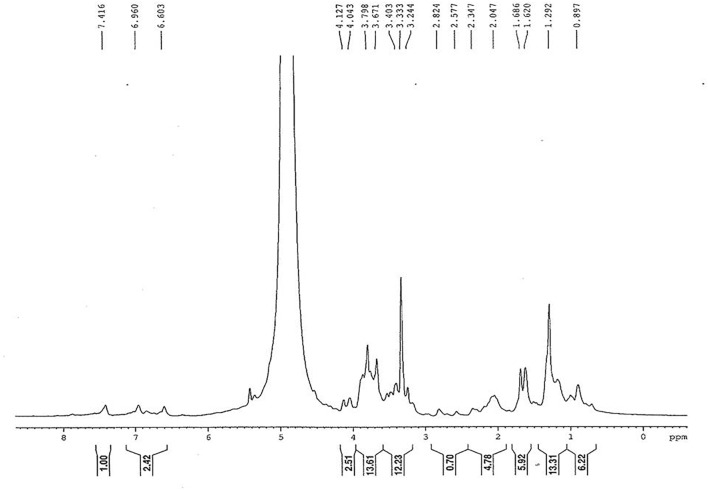
^1^H-NMR spectrum of *Cymbopogon citratus* (methanolic extract).

^1^H-NMR is effective technique for metabolites study in plant extract, as it analyzes all the metabolites present in extract (Kim et al., 2010; Heyman and Meyer, 2012). Figure 4 illustrated the ^1^H-NMR spectrum of *C. citratus* extract. Chemical shift obtained from ^1^H-NMR spectra can be due to proton on carbon or proton on Oxygen/Nitrogen. Chemical shift procured from proton on carbon is shown by signal at δ0.897,1.292 and 7.416 ppm which correspond to methyl, methylene and aromatic groups, respectively. The value range between δ 2–2.3 shows the presence of carbonyl group. Single range between δ4.043–7.416 and δ0.5–5 due to the shift for proton on Oxygen/Nitrogen which account for the presence of alcohol in the extract. Occurrence of allylic group in extract, is due to the shift acquired between δ 1.686–2.047. The Peak Fabrics obtained form NMR profile illustrated that *C. citratus* extract comprising of mixture of terpenoids and flavanoids.

### GC-MS Analysis of *C. citratus*

**FIGURE 5:**
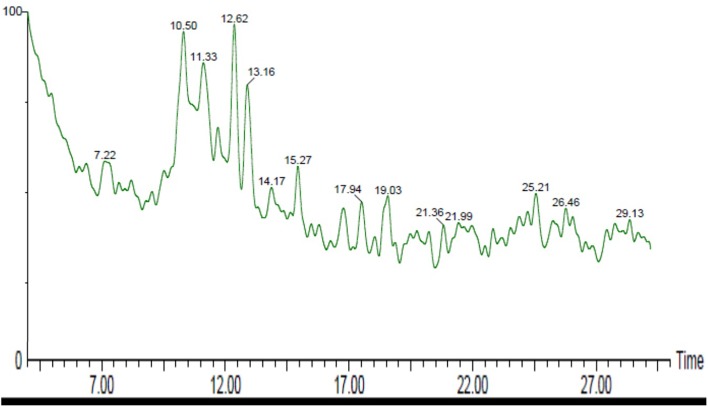
GC-MS chromatogram obtained from *Cymbopogon citratus* (methanolic extract); depicted the molecule peak eluted at particular retention time.

**FIGURE 6:**
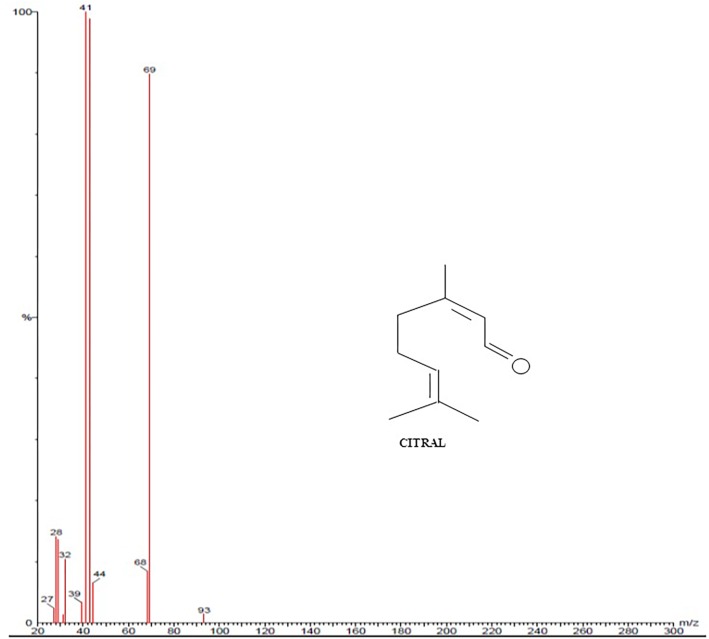
Illustrated the fragmentation pattern of Citral molecule present in *Cymbopogon citratus* (methanolic extract).

GC-MS was performed to validate the data acquired from FTIR and NMR of *C. citratus*. GC-MS spectroscopy has been widely used as a powerful structural characterization technique. Analysis of *C. citratus* extract revealed the presence of α-citral in the sample showed by peak eluted at 10.50 min (Figure 5). By observing system library peaks eluted with retention time of 12.62 and 13.16, corresponds to myrcene and farnasol. The fragmentation patterns of the peaks and identified compounds of the plant were shown in Figure 6, 7. By analyzing the fragmentation pattern of GC-MS divulged the existence of Citral, Myrcene, Farnesol, β-myrcene and β –Pinene with peaks at 29, 41, 69, 79, 93,105, and 119 m/z, respectively. Due to the hemolytic alpha and beta cleavage, citral shows peak at m/z 29, 69 (https://webbook.nist.gov/chemistry/) According to Nanjundaswamy et al. (2007), Kumar (2012), Madivoli et al. (2012), and Olorunnisola et al. (2014) bioactive component as elucidate above shows antibacterial effect. FTIR and NMR data indicated the presence of terpenoid and favanoids character of bioactive component, which was further proved by GC-MS data.

**FIGURE 7:**
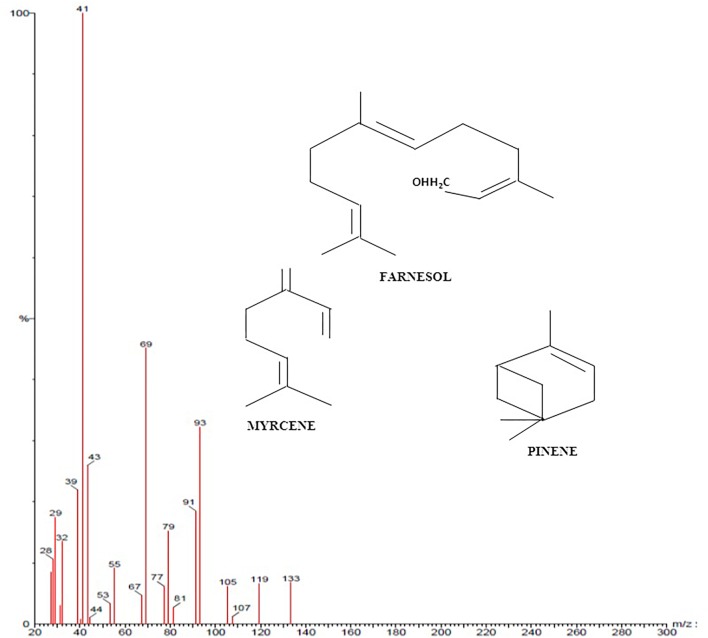
Illustrated the fragmentation pattern of Farnesol, Myrcene, and Pinene molecule present in *Cymbopogon citratus* (methanolic extract).

### XRD and EDXRF of CNPs

**FIGURE 8:**
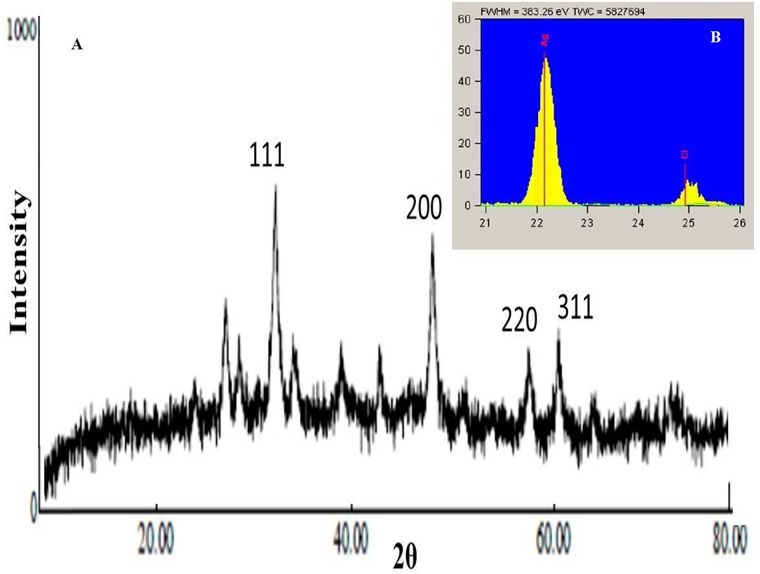
**(A)** XRD and **(B)** EDXRF graphs depicted the crystalline nature of CNPs with ∼ 35 nm.

XRD examination was executed to affirm the crystalline nature of the synthesized CNPs. Figure 8A delineated the XRD pattern of dried lyophized powder CNPs; four peaks 37.6°, 43.5°, 63.6° and 73.5° were obtained at 2θ, in the range of 20–80°. Braggs reflections obtained in this range clearly indicates that biosynthesized CNPs showed characteristic peaks which corresponds to the crystalline planes of face centered cubic structure, i.e., 111, 200, 220, and 311 (Oves et al., 2017; Qayyum et al., 2017). In addition, the weaker signals were recorded possibly due to components from the organic moieties present within the synthesized CNPs (Goudarzi et al., 2016). According to Zhang et al. (2016) size of silver nanoparticles lies between 2 and 100 nm. Nano-crystallite size can be calculated using Debye-Scherrer equation (Goudarzi et al., 2016; Jyoti et al., 2016):

$$D=\frac{K\lambda }{\beta Cos\theta }$$

Where D: Size of crystallites (nm), K: Crystallite shape factor (0.9), λ: X ray wavelength, β: Full width at half the maximum (FWHM) and θ: Braggs’ angle. Using Debye-Scherrer equation it was observed that the size of CNPs were ∼35 nm.

For understanding the elemental composition present in CNPs, EDXRF analysis was conducted (Shameli et al., 2012). In the present investigation EDXRF of CNPs (Figure 8B and Supplementary Figure S2) revealed the presence of silver (Ag) and chloride (Cl) where, silver (Ag, 87.1%) as the major constituent element compared to chloride (Cl, 12.9%). EDXRF reading proved that the required phase of silver (Ag) was present in the CNPs.

### Determination of Bactericidal Effect of *C. citratus* (CNPs) Through Antibacterial Assay and SEM Analysis

For the assessment of CNPs potential against test organisms; agar disk diffusion, minimum inhibitory concentration (MIC) and Minimum bactericidal concentration (MBC) techniques were performed. From the experimental range of CNPs (C_25_- C_150_μg/ml), C_25_ (μg/ml) concentration was determined to be the MIC for all bacterial species (*B. cereus, B. licheniformis, P. aeruginosa* and *E. coli*) while C_25_ μg/ml (*B. cereus, B. licheniformis)* and C_50_ μg/ml (*P. aeruginosa* and *E. coli*) as a MBC (Table 3). Investigation done by Hindumathy (2011) and Jafari et al. (2012) reported the MIC and MBC of *C. citratus* extract ranged between 50 and 150 mg/mL and 25 to 200 mg/mL, respectively. Tobramycin belongs to a class of drugs known as aminoglycoside antibiotics which is used to prevent or treat a wide variety of bacterial infections (especially related to eyes). According to Naik et al. (2010) investigation *B. cereus, E. coli*, and *P. aeruginosa* showed resistant behavior against tobramycin. While working on alkalinized *C. citratus* silver nano-particles Ajayi and Afolayan (2017) recorded the MIC values, ranged between 31.25 and 62.5 μg/ml and for antibiotic ciprofloxacin 31.25–15.63 μg/ml against *B. cereus, E. coli, E. faecalis, S. Flexneri.* Agar disk-diffusion data revealed that C_150_ (μg/ml) concentration expresses the maximum zone of inhibition (calculated in mm), for all bacterial species; *B. cereus* (20.12 ± 0.42), *B. licheniformis* (22.34 ± 0.4), *P. aeruginosa* (35.23 ± 0.46) and *E. coli* (31.87 ± 0.24) (Table 4 and Supplementary Figure S3). According to Zulfa et al. (2016) report, illustrated the maximum zone of inhibition of *Cymbopogon citratus* extract against *B. Cereus* and *E. coli* was 12.00 ± 1.41 and 7.50 ± 0.71, respectively. While comparing the CNPs results with previous studies, effective results of CNPs were reflected.

**Table 3:** MIC and MBC values of CNPs synthesized from *C. citratus*.

S. No.	Test organism	Positive control	Negative control	MIC and MBC CNPs (μg/ml)	
				
				MIC	MBC
1	*Bacillus cereus*	**+**	**-**	25	50
2	*Bacillus licheniformis*	**+**	**-**	25	50
3	*Escherichia coli*	**+**	**-**	25	25
4	*Pseudomonas aeruginosa*	**+**	**-**	25	25


*(-): Clear solution, (+): Turbidity.*

**Table 4:** Mean value of zone of inhibition of CNPs synthesized from *C. citratus* through agar disk-diffusion method.

Test organism	Zone of inhibition (mm)				
	
	CNPs (μg/ml)				
	
	AgNO_3_ (25 μg/ml)	C_25_	C_50_	C_100_	C_150_
*Bacillus cereus*	0	1.41 ± 0.12	6.93 ± 0.08	12.23 ± 0.4	20.12 ± 0.42
*Bacillus licheniformis*	0	1.92 ± 0.07	7.52 ± 0.42	13.24 ± 0.12	22.34 ± 0.4
*Escherichia coli*	0	2.51 ± 0.44	8.12 ± 0.12	18.96 ± 0.46	31.87 ± 0.24
*Pseudomonas aeruginosa*	0	2.91 ± 0.12	9.12 ± 0.14	19.23 ± 0.24	35.23 ± 0.46


*±: Standard Deviation (SD) and C_(25-150)_: range of CNPs concentration and AgNO_3_ (25 μg/ml): as a control. Mean values of each rows are significantly differ from each other according to statistical one way ANOVA (*P* ≤ 0.05).*

**FIGURE 9:**
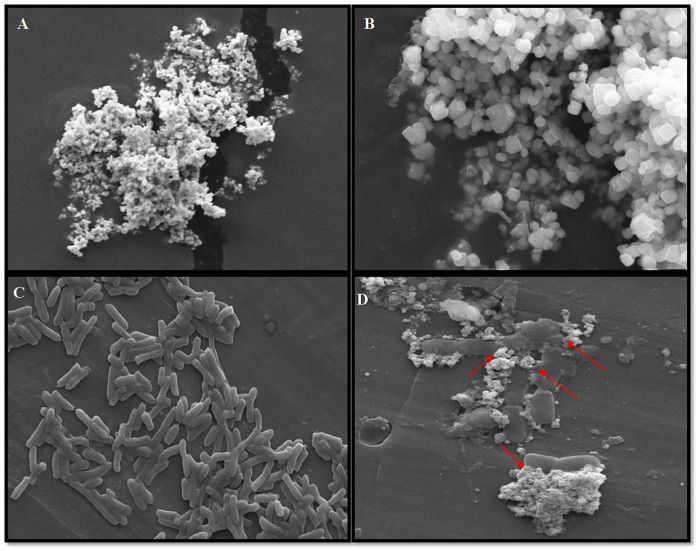
**(A)** Scanning micrograph of CNPs (with bar of 2 μm). **(B)** Depicted cuboidal structure of CNPs (with bar of 1 μm). **(C)** Represented untreated bacterial population with intact cell membrane (with bar of 1 μm). **(D)** Detrimental effect of CNPs on bacterial cell membrane were observed marked by red arrows (with bar of 1 μm).

Bactericidal profile of CNPs can be inspected by SEM studies, which is considered as the paramount technique for visual examination of bacterial interaction with synthesized nanoparticles as depicts in Figure 9. SEM images of CNPs illustrated the surface morphology in terms of size (20–40 nm) and shape (cuboidal) was represented in Figure 9A,B. Hong et al. (2016) illustrated the importance of cube shaped silver nanoparticles in antibacterial activity. Their study demonstrated that, while comparing the typical shaped silver particles that is nanosphere, nanocubes and nanowires; silver nanocube showed the strongest antibacterial activity as they can establish the close contact with bacteria due to the granulated shape and large surface area. Figure 9C represents untreated rod shape, diplo-bacillus intact bacteria with no sign of damage in cell wall. After treatment with CNPs, bacterial lysis was noticeably observed as marked with red arrows in Figure 9D. The capability of CNPs for antibacterial action is because of the diminished sizes of silver acting as capping agents with bioactive components present in plant extract. Researchers reported several approaches to describe the destructive nature of silver nanoparticles. Literature revealed that silver particles can interact with sulfur and phosphorous groups present in bacteria; continuous interaction causes the disruption of one of the most incumbent process, i.e., DNA replication which eventually ruptured the microbial structure and finally leads to the death of bacteria (Yugal et al., 2017). According to Xia et al. (2008) accretion of nanoparticles on bacteria showed the bactericidal effect, due to development of highly reactive species, i.e., ROS (Reactive oxygen species) which further leads to the formation of hydroxyl radicle and singlet oxygen species that disrupt the cell-wall and exterminated the bacteria (Lavakumar et al., 2015). Nevertheless, the factual operation behind lethality of silver nano-particle is not yet fully understood.

### Cytotoxicity Assay

**FIGURE 10:**
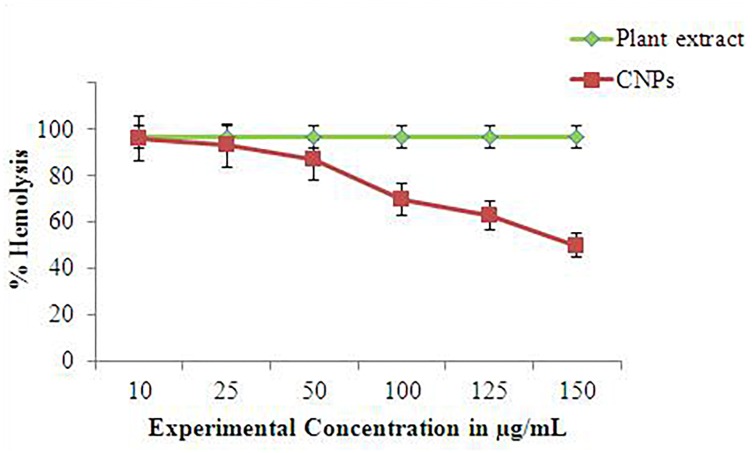
Graph elucidated the cell cytotoxicity of CNPs in terms of percentage of hemolysis.

Cytotoxicity analysis provided the significance in identifying the lethality of synthesized silver nanoparticle. Though, plant based nanoparticles are trending subject among researchers due to its broad spectrum effect on microorganism (Ovais et al., 2016), but its toxicity need to be classified (Yugal et al., 2017). According to the present data of *C. citratus* extract and CNPs, revealed that CNPs has more efficacious effect in hemolytic action than *C. citratus* extract Figure 10. For the present experiment 10–150 g/ml range (Plant extract and CNPs) was used. Data exhibited that increasing the concentration of CNPs has negative effect on RBCs. Several factors are responsible for this significant higher rate of hemolytic action of CNPs, one possible explanation of hemolytic action on RBCs was considered to be the release of oxidative stress products which further damage the membrane and caused for lots of noxious effect such as morphological alterations and hemagglutination (Huang et al., 2016). Increased surface area of CNPs due to their nano size was an another explanation for toxicity, which generates pore in surface of RBC and finally leads to the death of cells. Hong et al. (2016) study illustrated that smaller AgNPs (silver nanoparticles) with larger surface area shows detrimental effects by damaging the entire cells, as they can developed the close contacts with bacterial cells. Whereas selected concentration range of plant extract has no reaction on experimental cells, Costa (2015) cited the extract of *C. citratus* act as a therapeutic agent due its innocuous and anti-inflammatory nature after oral and tropical administration (*in vivo*) in rats (as an experimental animal). Finding of Rao et al. (2009) also suggested the antigenotoxic and radioprotective potential of *C. citratus* extract.

## Conclusion

Emerging resistant pathogenic species developed the interest in plant based nano-particles. In this study we demonstrated the elevated antimicrobial activity of synthesized *C. citratus* silver nanoparticles, i.e., CNPs against the gram positive (*B. cereus, B. licheniformis*) as well as gram negative (*P. aeruginosa, E. coli*) pathogenic species. Further Phytochemical, FTIR, NMR and GC-MS analysis helps to explore the bioactive components of *C. citratus* extract which helps in reduction of silver ion (Ag^+1^ to Ag^0^) and act as an capping agent. Data obtained from XRD, EDXRF and SEM revealed the structural and chemical properties of CNPs. In addition, SEM images evidenced the physical interaction between the CNPs and bacteria which indicated the detrimental damages caused by CNPs by rupturing the bacterial membrane. Antibacterial assay was conducted to determine the MIC and MBC values against the test organism. Moreover, cell cytotoxicity findings revealed that increased concentration of CNPs over plant extract has lethal effect on cells. Taken all together, this investigation demonstrates the successfully synthesizes potentially active CNPs which can use as an effective bio-medical application against pathogenic species.

## Author Contributions

PB carried out all the experiments. PB and ML wrote the manuscript. ML critically reviewed and edited the manuscript. Financial support was provided by ML, AA, and BL.

## Conflict of Interest Statement

The authors declare that the research was conducted in the absence of any commercial or financial relationships that could be construed as a potential conflict of interest.
