# Supplementary material for: Analytical Investigation of Cymbopogon citratus and Exploiting the Potential of Developed Silver Nanoparticle Against the Dominating Species of Pathogenic Bacteria
Source: Front Microbiol. 2019 Feb 27;10:282. doi: 10.3389/fmicb.2019.00282 (PMC6400849; doi:10.3389/fmicb.2019.00282)
Supplement: Supplementary file 1 [file Data_Sheet_1.pdf]

Supplementary Material

Analytical investigation of *Cymbopogon citratus* and exploiting the potential of developed silver nano-particle against the dominating species of pathogenic bacteria

PriyankaBasera, MeetaLavania*, Anil Agnihotri,BanwariLal.

The Energy and Resources Institute (TERI), New Delhi

*Correspondence:

Dr. MeetaLavania

meetal@teri.res.in

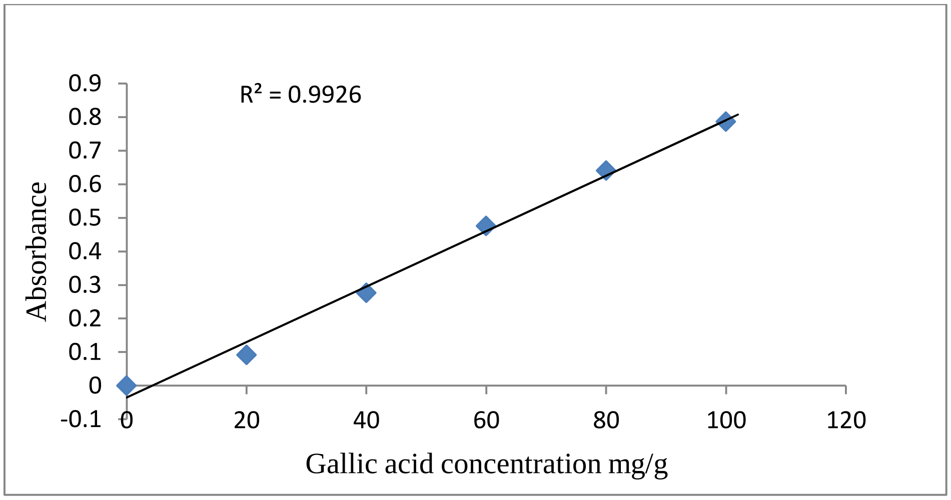

Figure S1 Folin-Ciocalteau gallic acid standard curve.

Table S1 Mean values of zone of inhibition in various plant extracts through agar disc-diffusion method.

Test organism	Zone of Inhibition (mm)					
	**Plants Extract (100 μg/ml)**					
	**A**	**B**	**C**	**D**	**E**	**F**
Bacillus cereus	1.2±0.12	2.2±0.6	3.5±0.12	1.4±0.11	1.3±0.1	10.23±0.08

Bacillus licheniformis	1.4±0.4	3.2±0.11	2.1±0.23	1.1±0.12	1.5±0.5	13.1±0.3
Escherichia coli	3.4±0.44	5.2±0.34	5.4±0.5	4.2±0.33	2.5±0.4	15.2±0.5
Pseudomonas aeruginosa	2.3±0.23	4.2±0.12	4.5±0.4	3.2±0.4	3.6±0.6	18.0±0.12

Note: ±: Standard deviation. A (*Phyllanthus emblica*), B (*Azadirachta indica*), C (*Capsicum annuum*), D (*Sapindus mukorossi*), E (*Plumeria obtuse*), F (*Cymbopogon citratus*). According to statistical one way Anova ($P \leq 0.05$), mean values of rows differ from each other.

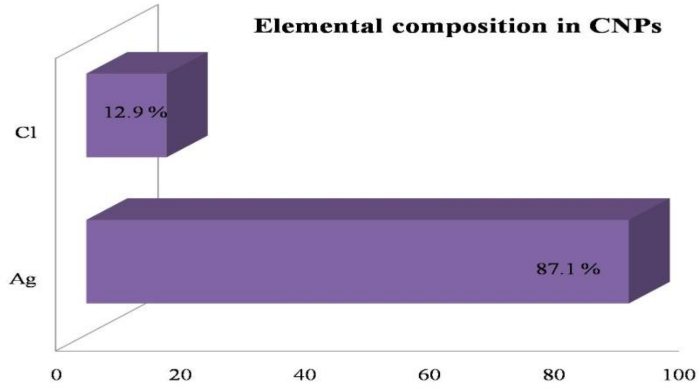

Figure S2. EDXRF graph illustrated the presence of elemental (Ag and Cl) composition in CNPs

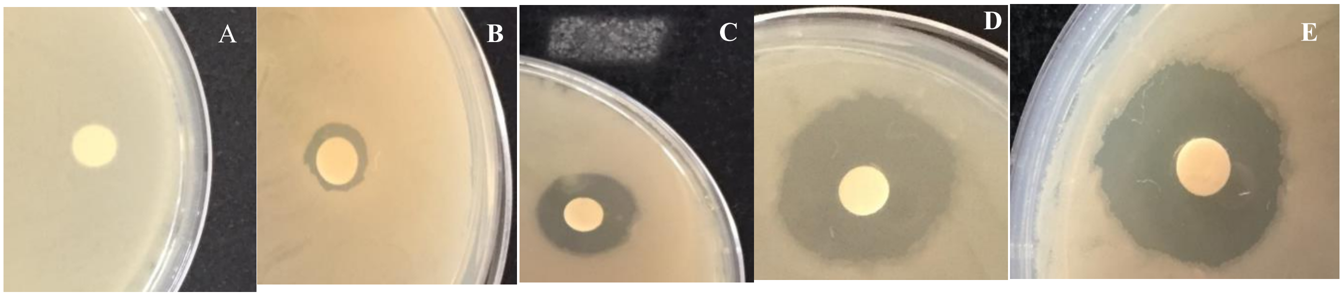

Bacillus cereus

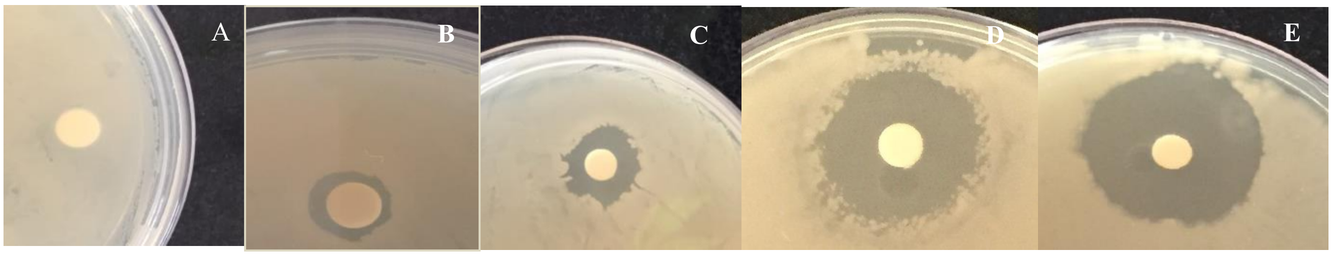

Bacillus licheniformis

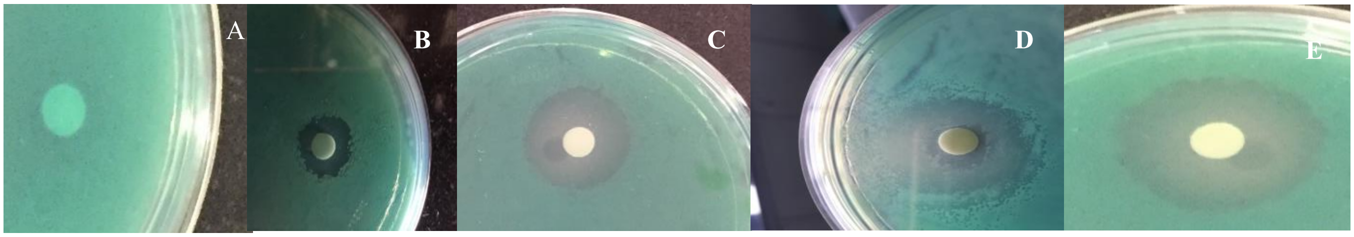

Pseudomonas aeruginosa

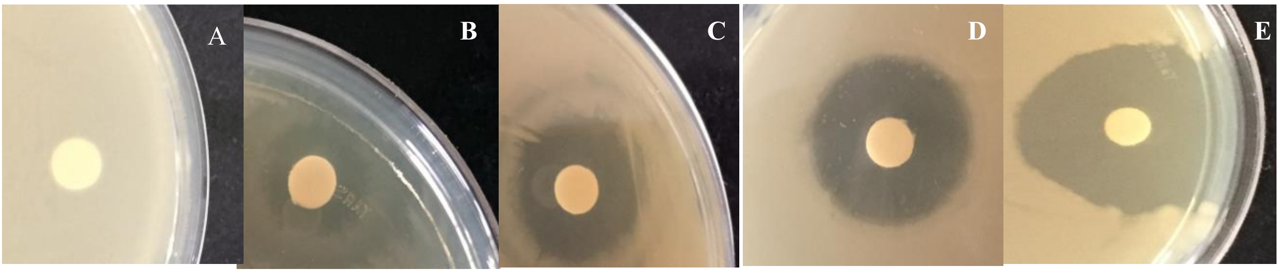

Escherichia coli

Figure S3 Depicted the zones of inhibition of CNPs using agar disc-diffusion method in a range of C_{25}-C_{150} µg/ml concentration. Where a) Control ($AgNO_3$ 25 µg/ml) b) C_{25} µg/ml c) C_{50} µg/ml d) C_{100} µg/ml and e) C_{150} µg/ml
